# Ecology meets cancer biology: The cancer swamp promotes the lethal cancer phenotype

**DOI:** 10.18632/oncotarget.3430

**Published:** 2015-03-20

**Authors:** Sarah R. Amend, Kenneth J. Pienta

**Affiliations:** ^1^ Department of Urology, The James Buchanan Brady Urological Institute, Johns Hopkins University, Baltimore, MD

**Keywords:** ecosystem, autoeutrophication, selection, cancer hallmarks, lethal phenotype

## Abstract

As they grow, tumors fundamentally alter their microenvironment, disrupting the homeostasis of the host organ and eventually the patient as a whole. Lethality is the ultimate result of deregulated cell signaling and regulatory mechanisms as well as inappropriate host cell recruitment and activity that lead to the death of the patient. These processes have striking parallels to the framework of ecological biology: multiple interacting ecosystems (organ systems) within a larger biosphere (body), alterations in species stoichiometry (host cell types), resource cycling (cellular metabolism and cell-cell signaling), and ecosystem collapse (organ failure and death). In particular, as cancer cells generate their own niche within the tumor ecosystem, ecological engineering and autoeutrophication displace normal cell function and result in the creation of a hypoxic, acidic, and nutrient-poor environment. This “cancer swamp” has genetic and epigenetic effects at the local ecosystem level to promote metastasis and at the systemic host level to induce cytokine-mediated lethal syndromes, a major cause of death of cancer patients.

## INTRODUCTION

It has long been appreciated that cancer is a disease of the whole organism, requiring not only the proliferation of malignant cells but also the participation of host cells and signaling factors [1, 2]. Cancer results in inappropriate host cell recruitment and activity, deregulated cell-signaling and regulatory mechanisms, and ultimately failure of the organism as a whole. This has striking parallels to the frameworks of ecological biology: multiple interacting ecosystems (organ systems) within a larger biosphere (body), alterations in species stoichiometry (host cell types), resource cycling (cellular metabolism and cell-cell signaling), and ecosystem collapse (organ failure and death) (Table 1).

**Table 1:** Ecological characteristics applied to cancer biology

Characteristic	Ecology	Cancer biology
Biosphere	Earth	Patient
Ecosystem	Lake	Organ system
Species	Animals and plants	Cell types
Abiotic factors	Land or water	Extracellular matrix
Nutrient cycling	Biogeochemical cycling	Cell-to-cell signaling
Invasive species	Beavers, kudzu	Cancer
Biogas	Swamp gas or firedamp	Cytokine release
Ecosystem collapse	Mass extinction	Organ failure and death

In their landmark 2000 and 2011 articles, Hanahan and Weinburg proposed a theoretical framework of essential “hallmarks” or capabilities required by a tumor to become malignant [3, 4]. While the Hallmarks model provides a good framework and common nomenclature for the cancer research field, it does not describe the necessary external processes of selection that lead to the emergence of these traits. It is now well recognized that as cancer evolves and progresses, so too does the tumor microenvironment, thereby (as noted by Hanahan and Weinberg) “enabling primary, invasive, and then metastatic growth” [4–9]. Ecology principles describe the enabling characteristics of the tumor microenvironment, including the factors that promote selection of aggressive traits in malignant disease as well as the local and systemic impact of cancer cells on the native host.

Critical to the survival of an individual organism and the success of the species as a whole are its interactions with other species and with its habitat. The interactions among all the living organisms of a community and the associated abiotic environment form an ecosystem, a self-regulating unit that cooperates as a whole to maintain overall homeostasis [10, 11]. The structure of an ecosystem is governed by species stoichiometry, niche differentiation, environmental disturbances, and resource availability.

In humans, dozens of ecosystems cooperate to perpetuate the required functions necessary to support life. Each organ system requires a specific stoichiometry of cell types (“species”) that interact with each other and with the extracellular matrix (“abiotic factors”) to form a productive ecosystem. Like any ecosystem, the host microenvironment requires balanced nutrient cycling in the form of cellular signaling to respond dynamically to perturbations to the ecosystem. Typically, small disturbances result in a more robust ecosystem, such as immunity following viral infection. In contrast, the native ecosystem of the host microenvironment is unable to recover from the chronic acyclic nutrient cycling induced by an invading species: cancer.

### Homeostatic nutrient cycling in ecosystems

Central to the success of an ecosystem is complete and efficient nutrient cycling. Nutrients, or resources, are substances required for healthy growth, viability, and function of a species [10]. Nutrient cycling is the exchange of these resources from the abiotic nutrient pool to biotic producers and consumers and ultimately back into the nutrient pool to repeat the cycle [10]. Nutrient loss must be countered by nutrient gain to maintain high ecosystem productivity [10, 12]. The classic example of closed resource cycling is the nitrogen cycle: atmospheric nitrogen (environmental nutrient sink) is fixed by plants (producers) that are then ingested by animals (consumers). Finally, animal waste and dead organic material decays and nitrogen is released back into the nutrient pool.

Nutrient cycles are self-regulated by dynamic feedback mechanisms to maintain ecosystem-wide homeostasis. Within this framework, each species occupies a unique niche, the functional role that describes its resource use and how it requires and supports other components of the ecosystem. A robust ecosystem with diverse niche occupants is able to rapidly recover from disturbances including altered resource availability, physical changes in the habitat, or other changes to the environment such as flooding or fire if they are separated in physical space and in time [13]. While minor disturbances to an ecosystem allow for increased resiliency, large disturbances may be catastrophic, resulting in community disassembly and ecosystem failure. Rapid or continuous alterations to any node of a nutrient cycle overwhelm the native feedback mechanisms that regulate complete resource recycling.

### Acyclic nutrient cycling: Ecosystem engineering by invasive species

One widespread instigator of unbalanced nutrient cycling is the introduction of a non-native species into an ecosystem. Successful invaders are characterized by rapid growth, high reproductive rates, and phenotypic plasticity [14, 15]. Concurrently, invasive species decrease the individual fitness of native species, altering community structure and nutrient cycling, and ultimately destroying the native ecosystem. Cancer cells act as an invasive species within a host organ, disrupting the homeostasis produced and controlled by the normal host cell species [2, 4, 16].

By nature of their expansion into a new ecosystem, invasive species are inherently ecosystem engineers. Ecosystem engineers change their abiotic habitat and modify resource availability to other species through direct mechanical alteration (allogenic engineering) or by modifying themselves (autogenic engineering) [17]. For example, kudzu is an invasive autogenic engineering species. As it grows and climbs native vegetation, it introduces new habitats for small animals, displacing the native vegetative species. In addition, kudzu competes with native plant species for sunlight and soil nutrients, thus altering local resource recycling. Perhaps the most recognizable allogenic engineer is the North American beaver. Beavers physically alter the native habitat by clear-cutting the terrestrial habitat and building dams that stop water flow on a river. The resulting beaver pond is a result of a destroyed habitat, displacing native species that require flowing water or tree stands for survival, thus breaking the consumer-producer relationship and altering species stoichiometry by altering resource cycling. Other species that favor open water or non-shaded wetlands colonize the beaver-engineered habitat. Ultimately, this results in complete community restructuring and the destruction of the native ecosystem even as a new one evolves to replace it.

Invasive species alter nutrient recycling through multiple different mechanisms. Any perturbation to an ecosystem's community structure will influence nutrient cycling as critical nodes increase or decrease, thus inducing acyclic nutrient recycling. Discrete trait invaders may directly increase usable nitrogen levels for consumers by introducing a novel nitrogen-fixation ability or may decrease nitrogen fixation of native species by altering soil chemistry [18–21]. Invasive species with rapidly decomposed organic waste will inherently increase the levels of nutrients in the ecosystem, ultimately leading to eutrophication of the habitat [20, 22, 23]. This has long term effects on the species in the ecosystem as altered nutrient cycling influences the quality of subsequent generations.

### Acyclic nutrient cycling: eutrophication

**Figure 1:**
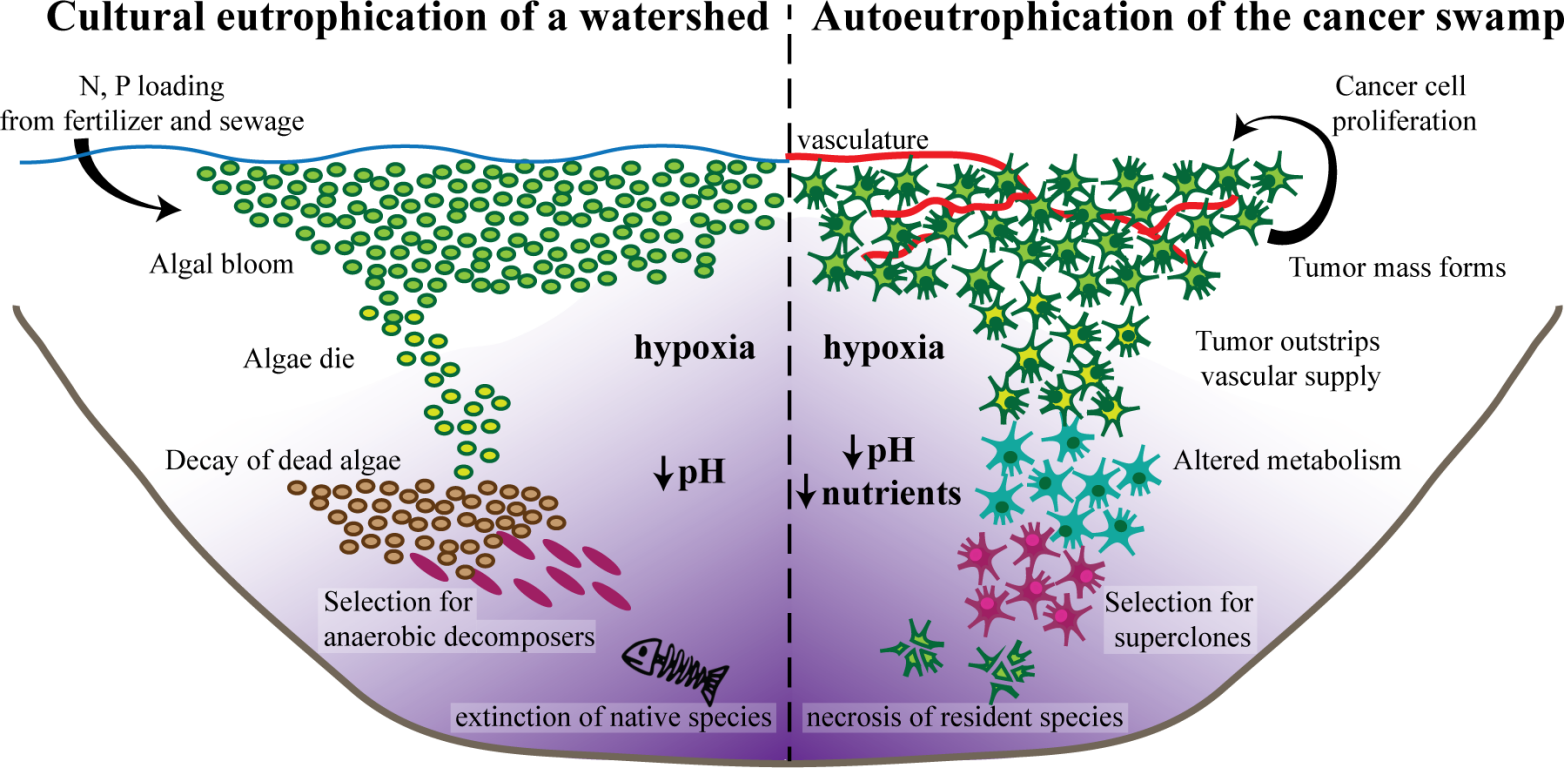
Autoeutrophication of the hypoxic, nutrient-poor, and acidic “cancer swamp” (Left) Excess nitrogen and phosphorus deposits stimulate the growth of photosynthetic algae, resulting in a characteristic algal bloom. As the algae die off, organic material accumulates and decomposition levels increase, leading to severe hypoxia. These harsh conditions select for efficient anaerobic decomposers. The build-up of the waste product of anaerobic fermentation, carbon dioxide, results in an acidic environment. Ultimately, the severe conditions lead to the local extinction of native species and eventual irreversible ecosystem collapse. (Right) Even in the absence of external stimuli, cancer cells have a high proliferation rate, rapidly expanding to a tumor mass analogous to an algal bloom. As the tumor grows, it quickly outstrips its vascular supply, resulting in a hypoxic microenvironment. To survive, the cancer cells alter their metabolism to utilize relatively inefficient anaerobic glycolysis, exhausting available nutrient sources. The accumulation of lactic acid, the waste product of anaerobic glycolysis, results in an acidic microenvironment. Ultimately, the harsh “cancer swamp” selects for highly lethal cancer superclones. Simultaneously, the toxic conditions lead to increased rates of necrosis, extinction of native host cell types, and eventual organ failure.

Eutrophication is the enrichment of an ecosystem with chemical or organic nutrients. Eutrophication is considered a healthy process when it occurs slowly on a geological time scale as part of the natural aging of a lake to a productive meadow [24]. When accelerated, however, eutrophication dismantles normal nutrient cycling and litter feedbacks, resulting in altered species stoichiometry and, if left unchecked, ecosystem failure [25]. Human acceleration of eutrophication (cultural eutrophication) of watersheds is one of the most apparent examples of forced acyclic nutrient cycling [10, 26]. Pollution in the form of fertilizers and sewage leads to local nutrient enrichment, specifically of phosphorus and nitrogen, two of the limiting growth factors necessary for photosynthesis [27]. The rapid accumulation of excess nutrients accelerates the creation of a swamp by inducing acyclic resource recycling that leads to the growth and expansion of photosynthetic organisms such as short-lived cyanobacteria that compose characteristic algal blooms. As the organic material of these algae accumulates, decomposition levels increase, consuming high levels of oxygen and leading to severe hypoxic conditions. The oxygen-poor environment is unable to support native consumer species such as fish or mollusks and is colonized by detritus-feeders (Figure 1). Nutrient cycling becomes weighted towards the activities of producers and lacks the negative feedback from consumer species. In addition, algae blooms may also directly poison consumer species, further exacerbating acyclic resource cycling [28, 29]. Such an ecosystem is unstable and susceptible to irreversible collapse.

**Figure 2:**
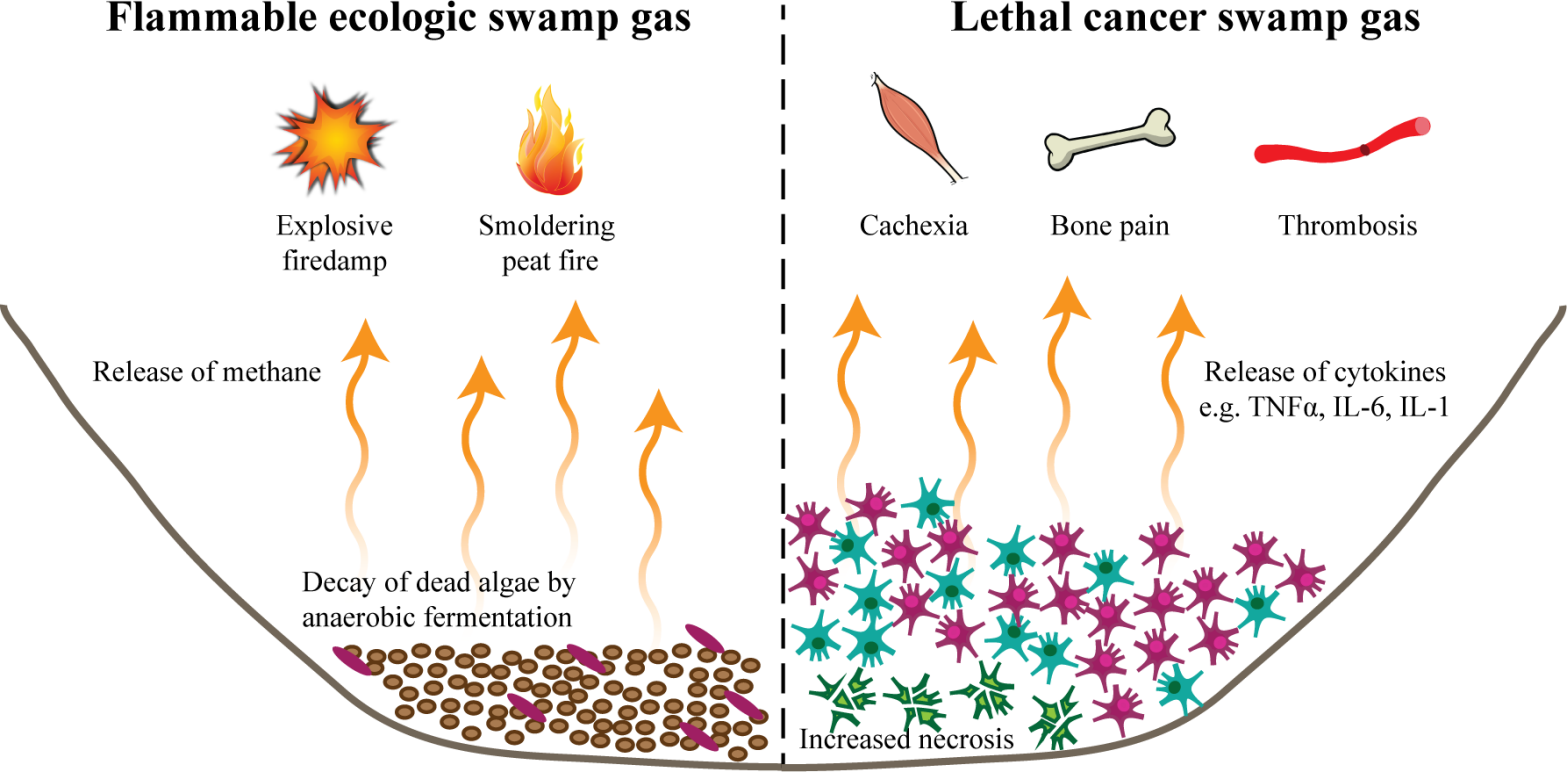
Release of toxic swamp gas (Left) A byproduct of decomposition by anaerobic fermentation is methane gas that bubbles to the surface as swamp gas. When near a sufficient ignition source, accumulation of this gas can lead to smoldering underground fires in peat fields or explosions in coal mines (“firedamp”). (Right) The release of lysed cell products of necrotic cells combined with the pro-inflammatory cytokines secreted from the cancer cells produce the equivalent of swamp gas. At high and persistent levels, the release of this “swamp gas” from multiple metastatic sites leads to cytokine-mediated smoldering (e.g. cachexia or bone pain) or acute (e.g. thrombosis) lethal syndromes, the cause of death in the many of patients.

The characteristic hypoxia of a eutrophic habitat requires anaerobic fermentation to decompose organic material. The byproduct of this process is typically a toxic combination of methane and hydrogen sulfide that bubbles to the surface as swamp gas. This phenomenon is largely harmless when gas is released into the atmosphere and diffuses, but in a closed habitat such as a peat bog or coal mine (“firedamp”) leads to dangerous accumulation of the flammable biogas, resulting in fires smoldering below ground or spontaneous explosions (Figure 2) [30, 31]. Thus, in a confined area, swamp gas can be rapidly catastrophic and may have far-reaching effects on neighboring ecosystems.

### Invasion and autoeutrophication: the “Cancer Swamp”

Cancer cells act as an invasive species as they expand within the host ecosystem as a primary tumor and again later as they colonize a distant site as a metastasis [9, 16]. Many of the hallmarks of cancer including high proliferation rates, avoiding predation by the native species, and resisting death are analogous to the high trait values expressed by ecological invasive species [3, 4]. With the advantageous phenotypic traits of an invasive species, tumor cells rapidly become ecosystem engineers by dismantling the host species community structure leading to the destruction of the native ecosystem and by generating a pro-tumorigenic microenvironment. Tumor cells alter the host ecosystem through both allogenic and autogenic means. As allogenic engineers, the cells physically alter the native habitat by secreting factors to destroy the extracellular matrix (production of MMPs), to recruit pro-tumorigenic macrophages (production of cytokines and chemokines including IL-6, TNFα, etc.), and to induce angiogenesis (production of VEGF) [3, 4, 32–34]. Simultaneously, autogenic engineering occurs as the tumor physically grows in size: tumor cells exhaust local sinks of energy and oxygen and increase litter concentration, ultimately overwhelming native feedback mechanisms.

This process, which we define as autoeutrophication, is analogous to ecological cultural eutrophication, but with significant differences. Most notably, while eutrophic watersheds are the result of external pollution, the “cancer swamp” is self-instigated. The positive feedback of high cancer cell proliferation rates and rapid tumor growth mean that the cancer cells act as both the “pollution” stimulus and responding “algal bloom” in the tumor microenvironment. Autoeutrophication, therefore, is self-driven by the cancer itself and, though it has substantial effects on the host, it is not governed by external factors (Figure 1).

As the cancer cells proliferate, the tumor rapidly grows and physically displaces the native habitat through autogenic engineering processes. Importantly, even in a clinically undetectable mass of 1 mm^3^, the tumor has outgrown the available vasculature, overwhelming both the incoming tributaries that provide oxygen and energy sources and the distuaries that carry away cellular waste [35]. Therefore, the tumor rapidly exhausts the local nutrient and oxygen sources while simultaneously poisoning the habitat with waste products. Under the hypoxic conditions characteristic of a poorly vascularized tumor, cancer cells switch from using oxidative phosphorylation for energy production to utilizing relatively inefficient anaerobic glycolysis, a process known as the “Warburg effect.” The corresponding accumulation of lactic acid, a waste product of glycolysis, results in an acidic microenvironment [36, 37]. Because glycolysis is relatively inefficient (2 mol ATP for each mol glucose) as compared to mitochondrial oxidative phosphorylation, energy sources are rapidly exhausted, resulting in a metabolite-poor habitat. Ultimately, this autoeutrophication displaces the native healthy ecosystem with a nutrient-poor, acidic, and hypoxic “cancer swamp” (Figure 1).

While a tumor mass directly promotes the destruction of the local ecosystem, the lethal clinical syndromes responsible for many cancer deaths are also the result of the toxic cancer swamp. Similar to algae blooms of a eutrophic lake, there are multiple simultaneous mechanisms of cancer cell-mediated toxicity. Analogous to the increased decomposition rates of algae, the hypoxic and glucose-depleted “cancer swamp” promotes cancer and host cell necrosis [38, 39]. In contrast with tightly regulated apoptotic cell death, necrotic cells expand, lose cell membrane integrity, and explode, releasing their intracellular contents into the environment. In addition, the deregulated “nutrient cycling” (cell-cell signaling) of the cancer cells within the “cancer swamp” leads to an accumulation of secreted cytokines and other factors. As with ecologic “swamp gas,” when low concentrations are released into open atmosphere, these factors are relatively harmless. However, at high levels within a closed system, such as the biosphere of a cancer patient, the release of “cancer swamp gas” is catastrophic, either inducing a smoldering lethal syndrome (e.g. cytokine-mediated cachexia or bone pain) or an acute lethal event (e.g. thrombosis) (Figure 2) [2, 40–45].

### Ecological inheritance: selection of traits by the self-engineered ecosystem of the cancer swamp

While ecosystem engineering describes an organism's impact on other species and communities of the ecosystem, the same actions also play a role in niche construction, i.e., how the environmental changes made by the species impact the selective pressure and subsequent adaptation of the species in its newly engineered niche [17, 46]. Inherent to the adaptive processes of niche construction is ecological inheritance. While genetic inheritance describes the genetic material inherited from an organism's ancestors, ecological inheritance describes the selective pressure associated with the engineered environment inherited from an organism's ancestors [47]. For example, when a beaver builds a dam, it not only is building a habitat, it also alters nutrient cycling and decomposition dynamics. Successful offspring, therefore, must be able to maintain and survive in the engineered environment by expressing high trait values for niche construction (dam building) and for traits favored in the niche (open-water pond and clear-cut wetland). Thus, there is a co-evolution of traits that generate the adaptive pressure (engineering) and traits that are dependent on the adaptive pressure (survival in the engineered ecosystem) [48].

As tumor cells engineer their environment to create the toxic “cancer swamp,” they simultaneously create a habitat that will exert selective pressure on subsequent generations of daughter cancer cells. Ecological inheritance of the cancer swamp may promote biodiversity within the cancer cell population, contributing to the high level of genetic and epigenetic heterogeneity within tumors [16]. However, the clonal architecture of cancer cells within a tumor suggests that most if not all cancer cells have the capacity to metastasize [49–51]. The stringent adaptive pressures of the malignant microenvironment of the “cancer swamp” may enrich for a small subset of highly lethal cancer cell clones that are phenotypically equipped to leave the primary ecosystem and migrate to and colonize a distant site (Figure 1). For example, hypoxia epigenetically induces the expression of the transcription factor HIF1α that in turn induces the expression of epithelial-to-mesenchymal transition (EMT) cellular programs that contribute to the production of the pro-metastatic mesenchymal phenotype [52–54]. Exposing murine sarcoma and melanoma cell lines to glucose starvation increases tumor foci in a forced lung metastasis model [55]. Moreover, culturing human cancer cell lines in acidic pH, the last characteristic of the “cancer swamp,” increases the mesenchymal phenotype *in vitro*, and increases metastatic potential *in vivo* [56–58].

The principals of ecology and ecological inheritance suggest that tumor cell heterogeneity can not only be generated by changes to the genome as a result of inherent genetic instability and altered DNA repair mechanisms but also by genetic trait selection as a result of environmental pressures in classic Darwinian fashion. Initiating events are likely the result of genomic alterations may be caused by many different events, including stochastic mutation and carcinogenic alterations to DNA damage repair mechanisms, among others [4]. Without the necessary selective pressures, however, the phenotypes that give rise to the additional necessary tumorigenic characteristics are unlikely to evolve. We hypothesize that the selective pressure of the “cancer swamp” provides the necessary selective pressure to direct natural selection to enrich for aggressive cancer cell clones with the phenotypic capacity to either survive in the harsh toxic “swamp” as an aggressive tumor cell or gain the ability to leave the tumor ecosystem and metastasize a distant site. Without the selective pressure of the engineered ecosystem, the cancer cells are much more likely to remain restrained to the primary tumor rather than metastasize to distant sites. This may explain, in part, why benign tumors exist. By growing extremely slowly, lipomas, for example, never outstrip their blood supply and therefore never feel the selective pressure to undergo the epigenetic changes that promote pro-metastatic cellular programs.

### Using restoration ecology strategies as anti-cancer therapies

Restoration ecology arose as a practical field of study in response to the increasingly negative impact of human activity on ecosystems worldwide. The preferred scenario for preservation is conservation, i.e. protecting the ecosystem prior to invasion or pollution. In cancer biology, these strategies are parallel to patient recommendations such as diet and exercise modification or daily administration of low-dose aspirin [59] to reduce general cancer risk.

**Figure 3:**
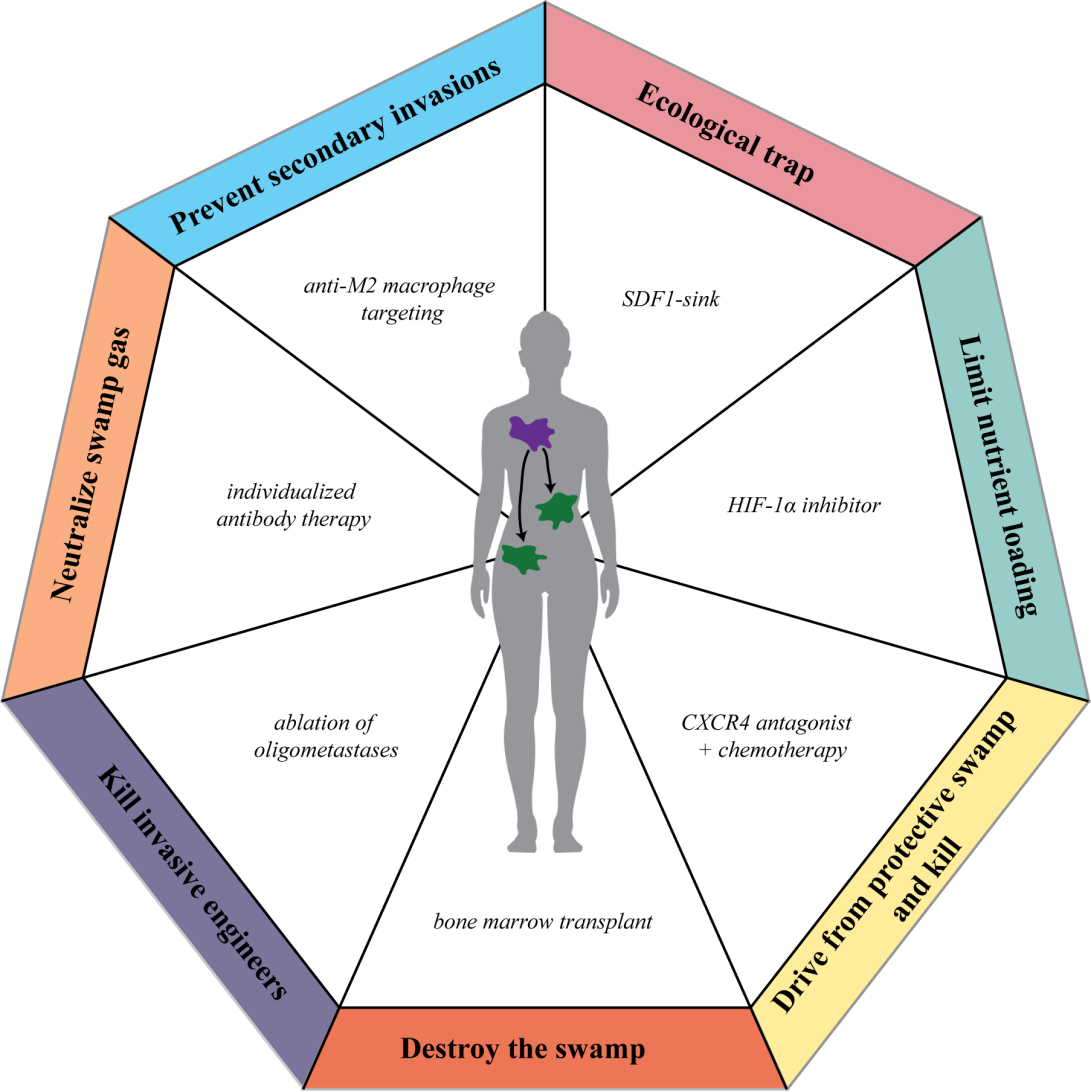
Using restoration ecology strategies as anti-cancer therapies Strategies used to restore damaged ecologic ecosystems can be applied to develop therapeutics to restore the “cancer swamp.”

Conservation efforts, however, while preferable both in ecology and in cancer biology, are often not sufficient on a larger scale and further intervention is necessary to restore the native ecosystem. Restoration ecologists aim to actively restore damaged ecosystems through systematic intervention to remove invading species, reduce eutrophication, and improve habitat quality for native species. Taking advantage of the successes of restoration ecology, such as the restoration of Lake Erie, cleanup efforts following oil spills, and community watershed and beach management, points of therapeutic intervention to likewise restore the “cancer swamp” can be identified (Figure 3, Table 2).

**Table 2:** Restoration ecology strategies applied to development of cancer treatments

Ecology		Cancer		
Problem	Intervention	Problem	Intervention	Clinical status
Ecosystem collapse due to ecosystem engineering	Draining the swamp	Cancer microenvironment replaces native ecosystem	Bone marrow transplant	Standard of care (multiple myeloma, leukemia)
Excessive nutrient loading	Reduce pollution	Cancer cell response to hypoxia	HIF-1α inhibitor	Pre-clinical [60–62]
Invasive species	Ecological trap	Cancer cell homing to metastatic site	SDF1-sink	Pre-clinical [65, 66]
Repeated invasions	Ecological trap and kill	Cancer cell homing to metastatic site	CXCR4 antagonist + chemotherapy	Phase 1/2 clinical trial [67]
Aggressive invasive ecosystem engineering	Kill dispersing invasive engineers	Oligometastases	Ablation of oligometastases	In use (radiation, surgery)
Secondary invasions of an unstable ecosystem	Physical barriers to prevent invasion	Recruitment of pro-tumor M2 macrophages	Anti-M2 macrophage targeting	Pre-clinical [71] Phase 1 clinical trial [70]
Release of toxic swamp gas	Preventative burn-off	Cytokine-mediated lethal syndromes	Individualized antibody therapy	In use as anti-inflammatory therapy

One of the largest successes of active intervention in both ecology and oncology is draining swampland. In regions with low-lying geography and high waterfall, swamps are the ideal breeding ground for malaria-carrying mosquitos. Historically, to reduce malaria in these regions, rather than directly killing the mosquitos using insecticides, a more effective strategy is to drain the swamp water and eliminate the favorable ecosystem for the mosquitos. The parallel strategy in hematological malignancies is total body irradiation followed by bone marrow transplant, leading to effective cures in leukemia and multiple myeloma patients.

The most effective management strategy to restore eutrophic watersheds — especially striking in the recovery of Lake Erie — is to reduce nutrient loading by implementing anti-erosion protocols, altering fertilizer application timing, and improving sewage treatment. In the autoeutrophic “cancer swamp,” because the eutrophication is self-instigated and self-maintained, reducing the equivalent of nutrient loading in the cancer microenvironment is currently not possible. Unlike in ecology, however, we can modulate the response of cancer cells to the eutrophic microenvironment with strategies such as HIF-1α inhibitors [60–62].

Ecological traps are poor-quality habitats that have disproportionate attractiveness for the actual survival value of the region [63]. Ecological traps can be used to attract otherwise harmful invasive species from the primary tumor site to a specific engineered site that can be easily managed [8, 64]. For cancer types that home to bone following the SDF-1 gradient, an artificial SDF1-sink could be introduced to attract malignant cells to an engineered site and specifically ablated [65, 66]. Using a similar strategy, resident invasive species can be driven away from their protective engineered habitat and eliminated in the vulnerable transit state. This strategy using CXCR4 antagonists in combination with chemotherapy has already completed clinical trials in leukemia [67] and is entering clinical trial for treatment of solid tumors.

A major concern in a region with a series of susceptible ecosystems is the dispersal of aggressive invasive ecosystem engineers from one site to additional sites, eventually leading to widespread ecosystem destruction. Immediate and specific elimination of these invasive engineers and their adopted environments, prior to secondary ecosystem collapse, is essential to protecting the larger region, especially after the primary invaded site has already undergone substantial engineering. In cancer biology, the equivalent problem is oligometastases, an intermediate state between a localized primary tumor and systemic metastatic disease [68]. Ablation of these small metastatic sites is becoming more widespread in clinical practice and has shown a survival benefit in a variety of solid tumor types [69].

Another concern of an invaded ecosystem is its susceptibility to secondary invasion by another species that may accelerate native ecosystem collapse. In ecology, physical barriers, such as the electric barriers to restrict Asian Carp entry into Lake Michigan, can be established to eliminate or reduce migration of secondary invaders into an engineered ecosystem. In cancer biology, one of the most abundant and destructive secondary invaders to a tumor site is pro-tumorigenic M2-macrophages. Targeted anti-M2 macrophage therapy to specifically eliminate these tumor-associated-macrophages has entered clinical trial and developing novel M2-targeting modalities is an active area of research [70, 71].

Finally, when active management to restore a damaged ecosystem fails, ecologists turn to physically managing the destructive element. For instance, controlled burning eliminates accumulated biogas to prevent mine explosions. Following a large-scale oil spill, a combination of mechanical, chemical, and biological methods are used to minimize ecosystem damage of the toxic oil on native fauna and flora. Similarly, in advanced cancer patients, individualized antibody therapy using therapeutics already in use such as anti-inflammatory agents for arthritis, psoriasis, and asthma, could be used to specifically neutralize a patient's specific cytokine “cancer swamp gas” to reduce cytokine-mediated syndromes (i.e. cachexia, bone pain, and thrombosis).

## SUMMARY

As cancer cells generate their own niche within the tumor ecosystem, ecological engineering and autoeutrophication displace normal cell function and result in the creation of a hypoxic, acidic, nutrient-poor environment. This “cancer swamp” has effects at both the local ecosystem and systemic host levels through multiple genetic and epigenetic mechanisms. The microenvironment is a protective habitat for the tumor cells, helping them to avoid detection and destruction by host immune cells that avoid the harsh conditions. Simultaneously, the “cancer swamp” attracts pro-tumorigenic immune cells such as M2-tumor associated macrophages that further help engineer the environment (secretion of MMPs), further promoting tumor growth and expansion of the “cancer swamp.” Environmental hypoxia induces neo-angiogenesis to increase circulation and therefore oxygen and nutrient levels within the tumor, in essence “irrigating” the “cancer swamp,” ensuring that the tumor does not undergo immediate ecosystem collapse. Hypoxia and acidity also promote EMT in cancer cells, increasing the likelihood of successful metastasis by enhancing motility and invasive phenotypes. At the host level, the combination of the release of lysed cell products as well as pro-inflammatory chemokines and cytokines from multiple metastatic sites leads to the production of the cancer equivalent of swamp gas, ultimately leading to a cytokine mediated death in many cancer patients.
